# Prevalence and Risk Factors for Hypoparathyroidism Following Total Thyroidectomy in Taif City

**DOI:** 10.7759/cureus.32460

**Published:** 2022-12-12

**Authors:** Rami F Algethami, Faisal Algarni, Shouq Fallatah, Rahaf A Almehmadi, Hatoon Aljuaid, Abdullah S Alsalem, Mohammad Eid M Mahfouz, Majed Alosaimi

**Affiliations:** 1 Medicine and Surgery, Taif University, Taif, SAU; 2 Medical School, College of Medicine, Taif University, Taif, SAU; 3 Medical School, Taif University, Taif, SAU; 4 Medicine and Surgery, College of Medicine, Taif University, Taif, SAU; 5 Otolaryngology - Head and Neck Surgery, Armed Forces Hospital, Ministry of Defense, Taif, SAU; 6 Surgery, Taif University, Taif, SAU

**Keywords:** saudi arabia, taif city, thyroidectomy, hypoparathyroidism, prevalence

## Abstract

Background

Postoperative hypoparathyroidism has been investigated in health records and surgical cohorts, but the results have been highly variable and imprecise. It is not clear how often endocrinologists encounter this hormone deficit in clinical practice. Thus, the aim of this study is to determine the incidence of permanent hypoparathyroidism and the factors associated with it in a group of post-thyroidectomy patients followed at three tertiary care institutions in Taif city.

Materials and Methods

A retrospective cohort analysis was done to examine patients who had a total thyroidectomy in the city of Taif between January 1, 2015, and December 31, 2019. Patients were eligible for the study if they received total thyroidectomy, were above the age of 18 years, had surgical and pathological data available, and had been monitored in the same institution for at least a year after their thyroidectomy. Patients who did not return for follow-up care following surgery were excluded from the study.

Results

The incidence of hypoparathyroidism was 10.3%, and females had a higher prevalence (12.1%) than males (3.2%). In patients with two and three parathyroid glands, hypoparathyroidism was found to be more prevalent (33.3% and 25.5%) in permanent histological sections. There was no single independent risk factor for hypoparathyroidism according to a logistic regression model.

Conclusion

The incidence rate of hypoparathyroidism following total thyroidectomy was about 10.3%. There were no independent risk factors identified for hypoparathyroidism after total thyroidectomy. Permanent hypoparathyroidism severely affects the quality of life, and research should be done to prevent its incidence after thyroidectomy.

## Introduction

The thyroid gland, consisting of two connected lobes, is one of the largest endocrine glands in the human body, weighing 20-30 g in adults. Thyroid lesions are often found on the gland, with a prevalence of 4-7%. Most of them are asymptomatic, and thyroid hormone secretion is normal [1].

Total thyroidectomy is a well-known surgical technique used to treat thyroid disorders. The occurrence of hypoparathyroidism, however, is one of the most significant risks of total thyroid surgery. Hypoparathyroidism is a rare endocrine condition characterized by low or insufficient levels of circulating parathyroid hormone (PTH), leading to hypocalcemia (low levels of serum calcium) [2]. Hypocalcemia can be acute with paresthesia and neuromuscular instability or chronic with seizures, cataracts, ectopic calcifications, abnormal teeth, abnormal renal function, and psychiatric illnesses [3]. Injury to the parathyroid glands at operation, by inadvertent removal, direct damage, or through disturbance of their blood supply, can cause hypoparathyroidism [4]. The most common cause of hypoparathyroidism is believed to be post-surgical procedures [1]. Post-surgical hypoparathyroidism is commonly classified as permanent or temporary. Permanent hypoparathyroidism is defined when a medical regimen is required for longer than 12 months [5]. Parathyroid failure following total thyroidectomy is now considered the most prevalent complication and the primary reason for hospital readmission following discharge.

In a previous study, the incidence of transient and permanent hypoparathyroidism is reported to be 27.4% and 12.1%, respectively, for patients undergoing thyroidectomy [6]. Postoperative hypoparathyroidism is commonly acknowledged to be a debilitating condition. Postoperative hypoparathyroidism is a surgical complication that may occur after any type of neck surgery for several clinical entities, mainly thyroid and parathyroid disease, and is the result of inadvertent parathyroid tissue damage during surgery, either by excision or compromised blood flow to the parathyroid glands. However, doctors are hesitant to accept the premise that parathyroid injury is mostly a technical issue because of their training and or experience [7]. Despite the fact that identifying and preserving parathyroid glands might be challenging, this cannot be used as an excuse for lack of experience or inadequate training. A combination of a surgeon's inexperience and a low volume of operations frequently causes technical errors. Even if patients' PTH levels are within the normal range following surgery, some patients may experience episodes of hypocalcemia and require replacement medication at any time. The parathyroid glands' reduced secretory capacity is thought to be the cause of the muted reaction to hypocalcemia following total thyroidectomy, resulting in a maladaptive response to low serum calcium levels [8].

Recent research has found several risk factors for parathyroid failure following total thyroidectomy, including Graves' disease, lymph node dissection, and caseload [3,9]. A prospective study conducted in Italy showed that the incidence of hypoparathyroidism was 28.8% [10]. A retrospective study conducted in Spain showed that the prevalence of hypoparathyroidism at discharge after total thyroidectomy is 48% [2]. Another study conducted in Jeddah showed that the prevalence of hypocalcemia on the second day after surgery was 67.4% of patients [11]. To our knowledge, there are no studies done in Taif city that determined the incidence of hypoparathyroidism after total thyroidectomy. Thus, our study aimed to assess hypoparathyroidism's prevalence and risk factors in patients undergoing total thyroidectomy in Taif city.

## Materials and methods

This was a retrospective cohort study conducted during the period from February 1, 2021, to January 31, 2022, on patients who underwent total thyroidectomy during the period January 1, 2015, to December 31, 2019, in three hospitals in Taif city (King Abdulaziz Specialist Hospital, Al-Hada Hospital, and King Faisal Hospital). The inclusion criteria for the study included (a) patients who underwent total thyroidectomy who are above 18 years old, (b) availability of surgical and pathological records, and (c) patients who followed up in the same hospital for at least one year after thyroidectomy. Patients with missing postoperative follow-up data were excluded from the analysis. This study was conducted in accordance with the Declaration of Helsinki (as revised in 2013). The Research and Ethics committee of Taif University approved the study, with approval letter number 491, dated 21/01/2021. Informed consent from patients was not required for this study because it was non-interventional and solely a retrospective examination of data from ordinary clinical practice.

The patients' demographic information, medical history, surgical details, histological data, and results of blood tests (such as calcium and PTH levels) were all compiled retrospectively. Clinical data were obtained at several points, including after discharge from the hospital following surgery and six months later during follow-up. Prior to surgery, venous blood was drawn to measure blood calcium and parathyroid hormone levels, which were then retested the following morning. 

Data management and statistical analysis

Data was entered on the computer using a single calibrated investigator's Microsoft Office Excel Software. An independent biostatistician transferred the data to SPSS Statistics v.23 for statistical analysis (SPSS Statistics for Mac OS, IBM Corp., Armonk, NY). Descriptive statistics in percentages and frequencies were used to present categorical data. Mean and standard deviation was used for describing continuous variables. Pearson's chi-square test was used as a test of significance for determining the relationship between categorical variables. A logistic regression model was conducted to assess risk factors for developing hypoparathyroidism following total thyroidectomy.

## Results

Our analysis included the characteristics of 155 patients who had undergone total thyroidectomy in Taif city. Patients from three hospitals were included in the study; 71 patients (45.8%) were from King Abdul Aziz hospital, 50 patients (32.3%) were from Al-Hada hospital, and 34 patients (21.9%) were from King Faisal hospital. Additionally, 87 (56.1%) of cases were treated by a consultant, and 124 (80%) were female patients. The baseline characteristics showed that 29 (18.7%) patients had one gland, 12 (7.7%) patients had two glands, four patients (2.6%) had three glands, and only one patient (0.6%) had all four glands, 29 (18.7%) had lymph node extension, 53 (34.2%) had malignant neoplasms, four (2.6%) had complications, 15 (9.7%) had low calcium level before surgery, and 11 (7.1%) had low PTH levels before surgery (Table 1). The mean age of the patients was 44.1± 11.4 years.

**Table 1:** Baseline characteristics of the cases PTH: parathyroid hormone

Baseline characteristics of the cases			
		Frequency	Percent
Hospital	Al-Hada	50	32.3
	King Abdulaziz	71	45.8
	King Faisal	34	21.9
Surgeon responsible for the procedure	Consultant	87	56.1
	Specialist	7	4.5
	Resident	11	7.1
	Not mentioned	50	32.3
Gender of patient	Female	124	80.0
	Male	31	20.0
Number of PTH glands seen in histopathological examination	1	29	18.7
	2	12	7.7
	3	4	2.6
	4	1	.6
	None	108	69.7
	No histopathology done	1	.6
Lymph node extension	No	125	80.6
	Yes	29	18.7
	Not mention	1	.6
Type of neoplasm	Benign	102	65.8
	Malignant	53	34.2
Number of surgeries done	One	153	98.7
	Two	2	1.3
Complications	No	151	97.4
	Yes	4	2.6
Calcium level before surgery	Low	15	9.7
	Normal	120	77.4
	Not recorded	20	12.9
Calcium level after surgery	Low	70	45.2
	High	1	.6
	Normal	83	53.5
	Not recorded	1	.6
Calcium level at discharge	Low	39	25.2
	Normal	107	69.0
	Not recorded	9	5.8
PTH level before surgery	Low	11	7.1
	High	4	2.6
	Normal	58	37.4
	Not recorded	82	52.9
PTH level after surgery	Low	31	20.0
	High	5	3.2
	Normal	48	31.0
	not recorded	71	45.8

The analysis showed the incidence of hypoparathyroidism was 10.3% (n=16). When we compared the prevalence of hypoparathyroidism with different baseline and perioperative characteristics, there were no statistically significant differences seen between the two age groups (p=0.927). The prevalence was seen more in females (12.1%) than males (3.2%) even though there were no statistically significant differences between the two genders. There were no statistically significant differences seen in the prevalence of hypoparathyroidism between patients who were operated on by different types of surgeons (p=0.906). The prevalence of hypoparathyroidism as a post-operative complication was found to be more among patients who had two and three PTH gland dissections (33.3% and 25.5% respectively)(p=0.045). There were no significant differences in prevalence observed for lymph node extension, type of neoplasm, number of surgeries performed, complications, calcium level before surgery, and PTH level before surgery (p>0.05) (Table 2).

**Table 2:** Hypoparathyroidism and its association with baseline characteristics PTH: parathyroid hormone

Hypoparathyroidism and its association with baseline characteristics					
		Hypoparathyroidism		Total	P values
		No	Yes		
Age	<40 years	45	5	50	0.927
		90.00%	10.00%	100.00%	
	>=40 years	94	11	105	
		89.50%	10.50%	100.00%	
Gender	Female	109	15	124	0.147
		87.90%	12.10%	100.00%	
	Male	30	1	31	
		96.80%	3.20%	100.00%	
Surgeon designation	Consultant	77	10	87	0.906
		88.50%	11.50%	100.00%	
	Not mention	46	4	50	
		92.00%	8.00%	100.00%	
	Resident	10	1	11	
		90.90%	9.10%	100.00%	
	Specialist	6	1	7	
		85.70%	14.30%	100.00%	
Parathyroid gland	1	29	0	29	0.045
		100.00%	0.00%	100.00%	
	2	8	4	12	
		66.70%	33.30%	100.00%	
	3	3	1	4	
		75.00%	25.00%	100.00%	
	4	1	0	1	
		100.00%	0.00%	100.00%	
	No	97	11	108	
		89.80%	10.20%	100.00%	
	No histopathology	1	0	1	
		100.00%	0.00%	100.00%	
Lymph node extension	No	112	13	125	0.944
		89.60%	10.40%	100.00%	
	Yes	26	3	29	
		89.70%	10.30%	100.00%	
	Not mentioned	1	0	1	
		100.00%	0.00%	100.00%	
Type of neoplasm	Benign	90	12	102	0.413
		88.20%	11.80%	100.00%	
	Malignant	49	4	53	
		92.50%	7.50%	100.00%	
Number of surgeries performed	One	137	16	153	0.629
		89.50%	10.50%	100.00%	
	Two	2	0	2	
		100.00%	0.00%	100.00%	
Complications during surgery	No	135	16	151	0.492
		89.40%	10.60%	100.00%	
	Yes	4	0	4	
		100.00%	0.00%	100.00%	
Calcium level before surgery	Low	13	2	15	0.673
		86.70%	13.30%	100.00%	
	Normal	109	11	120	
		90.80%	9.20%	100.00%	
	Not recorded	17	3	20	
		85.00%	15.00%	100.00%	
PTH level before surgery	High	3	1	4	0.583
		75.00%	25.00%	100.00%	
	Low	9	2	11	
		81.80%	18.20%	100.00%	
	Normal	52	6	58	
		89.70%	10.30%	100.00%	
	Not recorded	75	7	82	
		91.50%	8.50%	100.00%	

The above parameters were analyzed using a logistic regression model to determine the risk of hypoparathyroidism. None of the parameters were independently associated with hypoparathyroidism (Table 3).

**Table 3:** Logistic regression for hypoparathyroidism PTH: parathyroid hormone

Logistic regression for hypoparathyroidism				
	Odds ratio (OR)	95% CI for OR		p-value
		Lower	Upper	
Age >=40 years	1.052	0.316	3.504	0.934
Gender = female	4.538	0.53	38.844	0.167
Surgery by non-consultant	0.738	0.213	2.558	0.632
Malignant neoplasm	0.643	0.191	2.159	0.475
PTH gland seen in histopathology	1.402	0.422	4.654	0.581
Types of surgery = two	0	0	.	0.999
Complication see	0	0	.	0.999
Calcium level before surgery = low	1.171	0.206	6.65	0.859
PTH level before surgery = low	2.54	0.403	15.989	0.321
Constant	0.033			0.003

## Discussion

Hypoparathyroidism is one of the common complications after total thyroidectomy. During thyroid surgery, the blood supply to the parathyroid glands is affected and can be caused most of the time by direct damage to the parathyroid glands or accidental removal of healthy parathyroid glands during the procedure [12]. Though rarely fatal, permanent hypoparathyroidism can cause significant morbidity for the patient and higher expenses for the healthcare system. Our findings showed that the incidence of hypoparathyroidism was 10.3%. A study done by Ru et al. reported an incidence of 3.9% after six months of follow-up [13]. According to the literature available, the incidence of hypoparathyroidism after total thyroidectomy varies from 7% to 37% [14-16]. Mulita et al. [17] reported that 15.89% of patients who underwent total thyroidectomy had hypoparathyroidism. This variability in incidence could be due to sample size and the variety of methods used to define this complication. While it is suggested that parathyroid glands be identified during surgery, not all parathyroid glands can be spotted if the search is limited to those in the normal sites. When bleeding occurs, parathyroid glands become discolored and might be mistaken for thyroid, nodal, or adipose tissue. During a thyroidectomy, subcapsular parathyroid glands may be more difficult to locate and are thus more susceptible to removal [18]. The variation in the rate of persistent hypoparathyroidism between studies is due to the use of different definitions for parathyroid gland function. Our study used PTH ≤10 pg/mL and hypocalcemia as a criterion to define hypoparathyroidism after thyroidectomy. In a study done by Al-Dhahri et al., PTH ≥10 pg/mL and the absence of hypocalcemic symptoms were defined as recovery symptoms from hypoparathyroidism [19].

The accepted method for analyzing fine-needle aspirate (FNA) cytology is the Bethesda categorization system. Atypia of undetermined significance/follicular lesion of undetermined significance (AUS/FLUS), often known as Bethesda category III, is the most contentious category due to its heterogeneity and inconsistent reporting. The total risk of cancer for thyroid nodules in Bethesda classifications III-IV is between 15-40% [20].

Our study didn't show any independent risk factors significantly associated with hypoparathyroidism, which is similar to the findings of Ritter et al. [14], except that it identified parathyroid tissue present on pathology report as an independent risk factor for developing permanent hypoparathyroidism. Our study found that the incidence of hypoparathyroidism didn't show significant differences when compared between surgeons who performed thyroidectomies. It is reported that the risk of permanent hypoparathyroidism is 5% in the hands of an experienced thyroid surgeon who performs several thyroid surgeries. In children, the incidence rate of permanent hypoparathyroidism is higher than in adults, which may be because pediatric thyroid surgeons encounter fewer thyroid surgeries and thus are more inexperienced in performing such surgeries [21]. However, the risk of hypoparathyroidism even in the hands of experienced surgeons was found to be high in our study. New methods such as infrared light during thyroid surgery could reduce such complications during thyroidectomy [22].

In our findings, cases with lymph node extension didn't show a higher incidence of hypoparathyroidism. In a three-year retrospective analysis by Ru et al., it was found that male gender, combined lymph node dissection, the tumor diameter of the thyroid gland, a second operation, and preoperative hypocalcemia were independently associated with the incidence of postoperative hypoparathyroidism [13]. Lymph node dissection is often required in the case of malignant thyroid tumors. Studies demonstrate that dissecting lymph nodes in the central group during thyroidectomy can considerably increase the risk of direct injury to the parathyroid glands or interrupt the blood supply [23]. Evidence shows that females are more likely to develop hypocalcemia after thyroidectomy [24-26], leading to a higher prevalence of postoperative parathyroid failure [22]. Low calcium levels among females are linked to a lower circulating level of vitamin D after thyroidectomy [27]. There was no incidence of hypoparathyroidism noted in cases where they had some complications during surgery. Numerous studies have found that hospitals with a high volume of thyroid surgeries have decreased complication rates post-thyroidectomy [28, 29].

There are a few limitations to this study. Firstly, this was a retrospective analysis of cases from three institutions that utilized prospective data. Secondly, there could have been variations in the definition criteria of hypoparathyroidism, which could have been subjected to institutional bias. Thirdly, the sample size of our study was not large enough due to the relatively small number of thyroidectomies performed in these three hospitals, perhaps contributing to a failure to establish a causal relationship between incidence and many other risk factors. Our study used low blood calcium (more objective) and lower PTH levels to determine hypoparathyroidism. It should be stressed that in order to rule out hypocalcemia, we didn't use any subjective factors such as patient-related hypocalcemia symptoms.

## Conclusions

The incidence rate of hypoparathyroidism following total thyroidectomy was about 10.3%. There were no independent risk factors identified for hypoparathyroidism after total thyroidectomy. Permanent hypoparathyroidism severely affects the quality of life, and research should be done to prevent its incidence after thyroidectomy.
